# Aprepitant inhibits the development and metastasis of gallbladder cancer via ROS and MAPK activation

**DOI:** 10.1186/s12885-023-10954-8

**Published:** 2023-05-23

**Authors:** Xueyan Cao, Yang Yang, Wei Zhou, Yue Wang, Xue Wang, Xianxiu Ge, Fei Wang, Fangfang Zhou, Xueting Deng, Lin Miao

**Affiliations:** 1 Medical Center for Digestive Diseases, Second Affiliated Hospital, Nanjing Medical University, 121 Jiangjiayuan Road, Gulou District, Nanjing, Jiangsu China; 2 Department of Child Health Care, Children’s Hospital of Nanjing Medical University, Nanjing, China; 3 Burn and Plastic Surgery, Jiangsu University Affiliated Hospital, Zhenjiang, China

**Keywords:** Aprepitant, NK‐1R, ROS, NF-κB, MAPK, Gallbladder cancer

## Abstract

**Background:**

Aprepitant, as a neurokinin-1 receptor (NK-1R) antagonist, originally applied for curing chemotherapy-induced nausea and vomiting, has been reported to have significant antitumor effect on several malignant tumors. However, the effect of aprepitant on gallbladder cancer (GBC) is not clear yet. This study aimed to investigate the anti-tumor activity of aprepitant on GBC and the potential mechanisms.

**Methods:**

The NK-1R expression of gallbladder cancer cells were examined by immunofluorescence. MTT assay, wound healing and transwell migration assay were applied to detect the effect of aprepitant on cell proliferation, migration and invasion. Flow cytometry was used to detect the apoptosis rate. The effects of aprepitant on the expressions of cytokine were examined by real-time quantitative PCR and MAPK activation were detected via immunofluorescence and western blotting. Besides, xenograft model was established to investigate the effect of aprepitant in vivo.

**Results:**

Our results indicated that NK‐1R was markedly expressed in gallbladder cancer cells and aprepitant effectively inhibited the proliferation, migration and invasion. Furthermore, the apoptosis, ROS and inflammation response were significantly boosted by aprepitant in GBC. Aprepitant induced NF‐κB p65 nuclear translocationin and increased the expressions of p-P65, p-Akt, p-JNK, p-ERK and p-P38, as well as the mRNA levels of inflammatory cytokines IL-1β, IL-6 and TNF-α. Consistently, aprepitant suppressed the growth of GBC in xenograft mice model.

**Conclusion:**

Our study demonstrated that aprepitant could inhibit the development of gallbladder cancer via inducing ROS and MAPK activation, which suggested that aprepitant may become a promising therapeutic drug against GBC.

**Supplementary Information:**

The online version contains supplementary material available at 10.1186/s12885-023-10954-8.

## Introduction

Gallbladder cancer (GBC) is the most common primary malignant tumor of the biliary tract, which originates from the mucosal epithelium of the gallbladder and cystic duct [1, 2]. Due to its characteristics of high heterogeneity, insidious onset, and easy metastasis, most patients are in advanced stage at initial diagnosis [3]. The 5-year survival rate of patients with GBC is less than 5% and the overall prognosis is extremely poor [4]. Surgical resection remains the only treatment with curative intent for GBC [5]. However, only 10% to 30% of GBC patients are suitable for surgery, and the recurrence rate is high [6]. Furthermore, radiotherapy and chemotherapy do not show distinct advantages in GBC, which generally have defects including low sensitivity, poor efficacy and serious side effects [4, 7]. Therefore, it is of great significance to develop new therapeutic strategies for patients with GBC.

Neurokinin-1 receptor (NK-1R), a tachykinin receptor, combines specifically with substance P (SP), which belongs to the tachykinin family [8]. SP/NK-1R system is widely distributed in various tissues, involved in many physiological and disease processes like pain, inflammation and immune response [9–11]. Recent studies demonstrate that SP/NK-1R is highly expressed in numerous cancer types and plays a prominent role in the tumor formation and progression [12–15]. Increasing experimental evidence suggests that SP/NK-1R signaling pathway could participate in tumor proliferation, invasion, metastasis and angiogenesis [16–19]. We have reported that the activation of SP/NK‐1R can promote the tumorigenesis of GBC [20]. Consequently, SP/NK-1R may be a novel target for the treatment of gallbladder cancer.

As a highly selective non-peptide NK-1R antagonist, aprepitant is a clinical drug approved by the Food and Drug Association (FDA) for the treatment of chemotherapy-induced nausea and vomiting (CINV) [21]. In recent years, aprepitant exerts an anti-neoplastic effect in a range of human malignancies [22–24]. For example, in human osteosarcoma, blockage of NK-1R by aprepitant can inhibit cell growth both in vitro and in vivo [25]. Meanwhile, there is no evidence that aprepitant has significant side effects even at high doses [26, 27].

However, the effect of aprepitant on GBC remains unclear. Previously, our team has reported that NK‐1R is highly expressed in human gallbladder cancer and the activation of NK‐1R by SP could promote the tumorigenesis via the Akt/NF‐κB axis [20]. Based on the above evidence, it is interesting to investigate the role of aprepitant in gallbladder cancer. In this study, we aimed to confirm the anticancer effects of aprepitant both in vitro and in mouse xenograft. To our knowledge, this is the first experiment to explore that aprepitant can inhibit the proliferation, invasion and metastasis of gallbladder cancer, which provided evidence that aprepitant may become a potential therapeutic drug for GBC.

## Materials and methods

### Cell culture

Human gallbladder cancer cell lines GBC‐SD and NOZ cells were purchased from cell banks of Chinese Academy of Sciences (Shanghai, China). GBC‐SD and NOZ cells were cultured in RPMI 1640 medium (Gibco, United States), added with 10% fetal bovine serum (FBS) (Gibco, United States) and 1% penicillin/streptomycin(Thermo, United States). All cell lines were incubated at 37 °C in a humidified atmosphere with 5% CO_2_ according to the manufacturer's instruction.

### Drugs

A stock solution of aprepitant (MedChemExpress, United States) at the concentration of 100 mM, was attained through dissolving the compound in sterile dimethyl sulfoxide (DMSO) (Sigma-Aldrich, United States). Then it was diluted to different concentrations (6.25–100 μM), so that the final concentration of DMSO in the culture medium did not exceed 0.1% in all the treatments.

### Cell Viability Assay

Colorimetric MTT assay was used for examining cell proliferation and cytotoxicity. GBC‐SD and NOZ cells were seeded in 96-well plates (5 × 10^3^ cells/well) and incubated for 24 h. Aprepitant with different concentrations were added to each well, and cells were incubated for the desired time. Then MTT solution (20 μL) was added to each well and incubated for 4 h at 37 °C. Next, the medium was removed and DMSO (150 μL/well) was added to solubilize the formazan crystals for additional 15 min. The absorbances were read on a microplate reader (Bio-Rad Laboratories, United States) at 490 nm.

### Wound healing assay

Cells were seeded on 6-well plates and incubated until they reached a density of about 90–95%. A scratch wound was made by a sterile 10 μL pipette tip in the central area of each well. Subsequently, cells were washed twice with PBS and then cultured with aprepitant at the desired concentration. The wounded gaps were photographed by microscope at 0, 12, 24 and 36 h.

### Cell migration and invasion assays

Transwell migration assay and Matrigel invasion assay were performed using 24‐well transwell chambers. Cells were seeded to the upper chamber in serum-free medium, and the lower chamber was filled with 600 μL of culture media containing aprepitant and 10% FBS. After incubated at 37 °C for indicated time, cells were fixed with ice‐cold methanol for 20 min, and then stained with 0.1% crystal violet for 20 min. After washing and removing the nonmigrant cells from the upper face of the transwell membrane, cell images were captured in the fields of each filter under microscope. The only difference between migration and invasion assay was whether the upper surface of transwell film was precoated with Matrigel matrix (BD, United States).

### Cell apoptosis

The effects of aprepitant on cell apoptosis were analyzed with the Annexin V-FITC kit (Vazyme, China) according to the manufacturer’s instructions. GBC‐SD and NOZ cells were seeded in 6-well plates and incubated overnight. After treated with the required aprepitant concentration for dedicated time, the cells were harvested through trypsinization and re-suspended in 1 × binding buffer at a concentration of 1 × 10^6^ cells/ml. Then cells (1 × 10^5^) were incubated with 5 μL of Annexin V-FITC and 5 μL of propidium iodide (PI) for 15 min at room temperature in the dark, followed by addition of another 400 μL of 1 × binding buffer. Finally, samples were assessed by flow cytometry (FACScan; BD Biosciences, United States) within 1 h.

### ROS detection

The level of reactive oxygen species (ROS) production was examined by the cellular ROS detection kit (Beyotime, China) following manufacturer’s protocol. GBC‐SD and NOZ cells were incubated with 10 μM DCFH-DA for 20 min at 37 °C in dark. After washed with serum-free medium, the cells were treated with aprepitant for 20 min. The production of intracellular ROS under different treatments was observed under fluorescence microscope (Olympus, Japan).

### Mitochondrial membrane potential depolarization detection

JC-1 fluorescent probe (Beyotime, China) was used to detect mitochondrial membrane potential (MMP). GBC‐SD and NOZ cells were seeded in 6-well plates and cultured overnight. After treated with aprepitant for 60 min, the cells were stained with JC-1 for 20 min at 37 °C. After washing with buffer solution, we observed MMP depolarization in the cells and acquired images using fluorescence microscope (Olympus, Japan).

### Immunofluorescence

Cells were seeded on a glass slide and fixed in 4% paraformaldehyde. After washed twice with PBS, these slides were then permeabilized using 0.5% Triton X‐100 solution (BioFroxx, Germany), and subsequently blocked with 5% BSA. Next, the primary antibody, rabbit-anti NK-1R (Sigma-Aldrich, United States) or mouse-anti NFκBp65 (Cell Signaling Technology) were incubated overnight at 4℃. Then these slides were washed with PBS and then incubated with flour 594-conjugated goat anti-rabbit IgG or flour 488-conjugated goat anti-mouse IgG (Keygen Biotech, China). Nuclei were stained with DAPI (Beyotime, China). Images were photographed by fluorescence microscope (Olympus, Japan).

### RNA extraction and quantitative real‐time PCR

Total RNA was extracted from cells with TRIzol reagent, and reverse transcription was performed with reverse transcription kit (Thermo Fisher Scientific, United States) according to the manufacturer's instructions. The subsequent real‐time PCR was carried out with the SYBR® Green qPCR Kit (Vazyme, China). The expression of mRNAs was calculated using the comparative cycle threshold (ΔΔCT) method and normalized to β-actin to obtain the relative mRNA level of each target. The sequences of primers are listed in Table 1.

**Table 1:** The primer sequences used for real-time PCR

Target Gene	Primer sequence (5′-3′)
IL-1β	F: AGGCTGCTCTGGGATTC R: GCCACAACAACTGACGC
IL-6	F: AGACAGCCACTCACCTCTTCAG R: TTCTGCCAGTGCCTCTTTGCTG
TNF-α	F: CTCTTCTGCCTGCTGCACTTTG R: ATGGGCTACAGGCTTGTCACTC
MMP-2	F: ACCTGGATGCCGTCGTGGAC R: TGTGGCAGCACCAGGGCAGC
MMP-9	F: CTATTTCTGCCAGGACCGCTTC R: CACCTGGTTCAACTCACTCCG
β-actin	F: GGGACCTGACTGACTACCTC R: TCATACTCCTGCTTGCTGAT

### Western blotting

Total proteins were isolated from the cell lines or mice xenograft. Protein lysates were separated on SDS PAGE gels and then transferred to polyvinylidene difluoride (PVDF) membrane (Millipore, United States). The membrane was incubated in 3% bovine serum albumin (BSA) or 5% nonfat milk for 1 h at room temperature and then incubated with specific primary antibody at 4˚C overnight. After incubation with horseradish peroxidase (HRP)‐labeled secondary antibody for 1 h at room temperature, the membrane was visualized by an enhanced chemiluminescence kit and images were obtained using chemiluminescence western blot detection system (Bio-Rad Laboratories, United States). The intensity of the western blot bands was measured with Quantity One software (Bio-Rad, United States). GAPDH (Abcam, UK) was used as the control. Western blot antibodies p-P65, p-Akt, p-JNK, p-ERK, p-P38, caspase-3, cleaved caspase-3, caspase-9 and cleaved caspase-9 were purchased from Cell Signaling Technology. HRP-conjugated anti-rabbit and anti-mouse secondary antibody were purchased from Abclonal (Wuhan, China).

### In vivo xenograft study

The animal study was approved by the Ethics Committee of Nanjing Medical University (protocol code IACUC-2106005). All experimental procedures were performed in accordance with the guidelines for laboratory animal care and the study is reported in accordance with ARRIVE guidelines. Adult male BALB/c nude mice (aged 6–8 weeks, weighing 18 to 22 g) were purchased from the Qinglongshan Animal Center of Nanjing (Nanjing, China) and maintained under standard laboratory conditions. GBC‐SD cells (1.0 × 10^6^ cells per mouse) were subcutaneously injected into 12 nude mice. After ten days of tumor inoculation, mice were randomly divided into control group and aprepitant-treated group (6 mice per group). The control group received phosphate-buffered saline (PBS) and the aprepitant-treated group was injected intraperitoneally with aprepitant (10 mg/kg) every other day. Mice body weight and tumor volume were recorded every 3 days. Tumor volume was calculated by the formula: volume = length × width^2^ × 0.52. After 21 days of treatment, all mice were sacrificed. The tumors were excised and weighed. All mice were executed using the cervical dislocation method. Then the tumors were frozen in liquid nitrogen for molecular analyses.

### Statistical analysis

GraphPad Prism was used to conduct all statistical analyses. Data are indicated as means ± SEM. Statistical analysis was performed using the unpaired Students t-test or one‐way ANOVA. The criterion for significance was *P* < 0.05 for all comparisons.

## Results

### Aprepitant inhibits the proliferation of gallbladder cancerin vitro

**Fig. 1:**
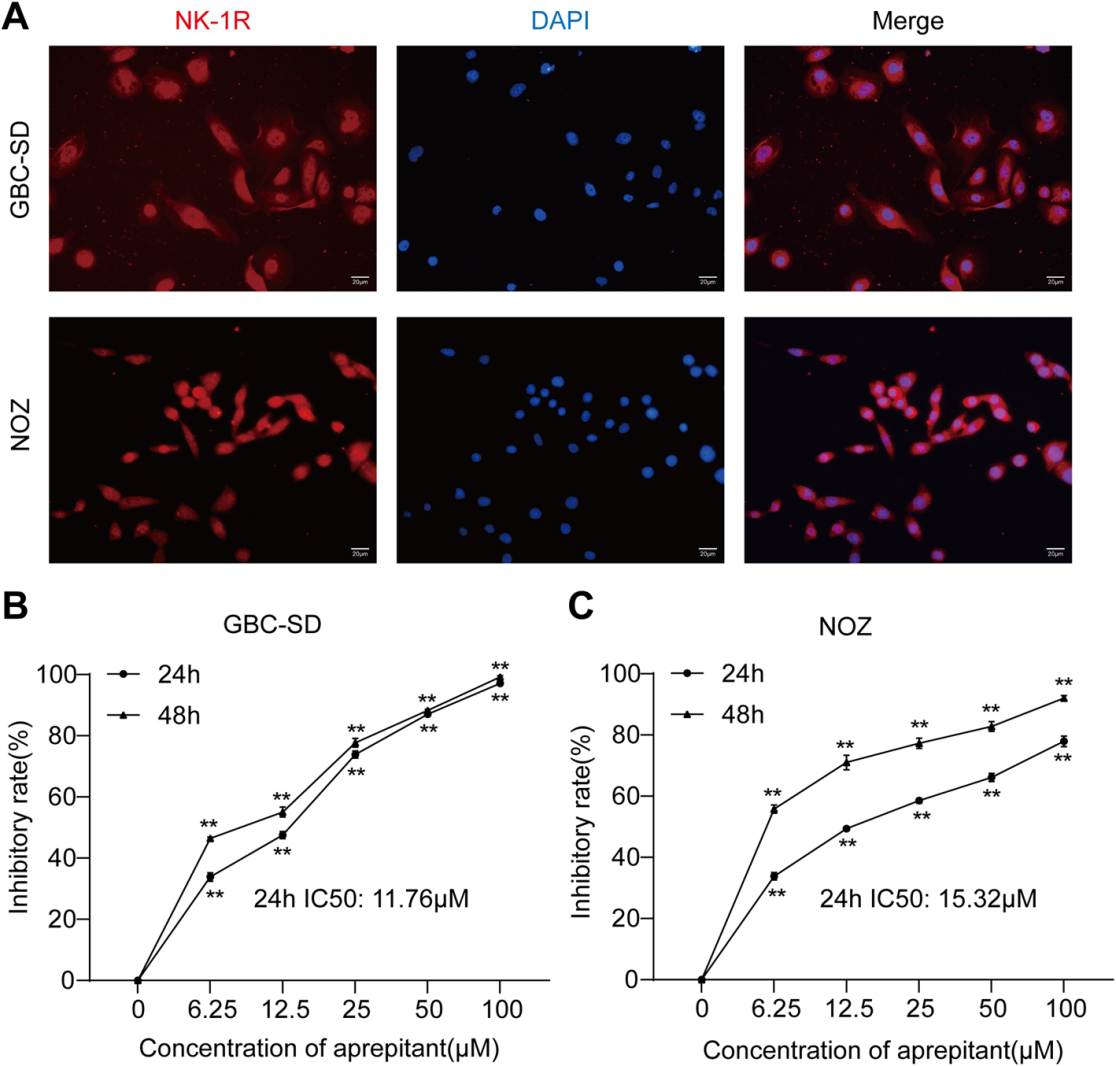
Aprepitant inhibited the proliferation of gallbladder cancer cells. Expression of NK‐1R was analyzed by immunofluorescence in GBC‐SD and NOZ cells (scale bar, 20 μm) (**A**). GBC‐SD cells (**B**) and NOZ cells (**C**) were treated with various concentrations of aprepitant (0 μM, 6.25 μM, 12.5 μM, 25 μM, 50 μM, 100 μM) for 24 h and 48 h. The cell viability was measured by MTT assay and the inhibition rate was calculated. Data are expressed as mean ± SEM (*n* = 6). One-way ANOVA. **p < 0.01, versus 0 h

We performed immunofluorescence to identify the expression of NK-1R in the human gallbladder cancer cell lines (GBC-SD and NOZ). As illustrated in Fig. 1A, the NK-1R was obviously expressed in GBC-SD and NOZ cells was examined. In order to assess the effect of aprepitant on GBC proliferation, GBC‐SD and NOZ cells were exposed to aprepitant at 6.25, 12.5, 25, 50, and 100 µM, respectively, and MTT assay was used to detect cell growth. Aprepitant markedly inhibited the cell proliferation in the Fig. 1B,C, which indicated that aprepitant has potential antiproliferative effects. As represented in IC50 calculation following 24 h of treatment, the IC50 value for GBC‐SD and NOZ cells were 11.76 and 15.32 µM respectively.

### Aprepitant restrains the migration and invasion of gallbladder cancerin vitro

**Fig. 2:**
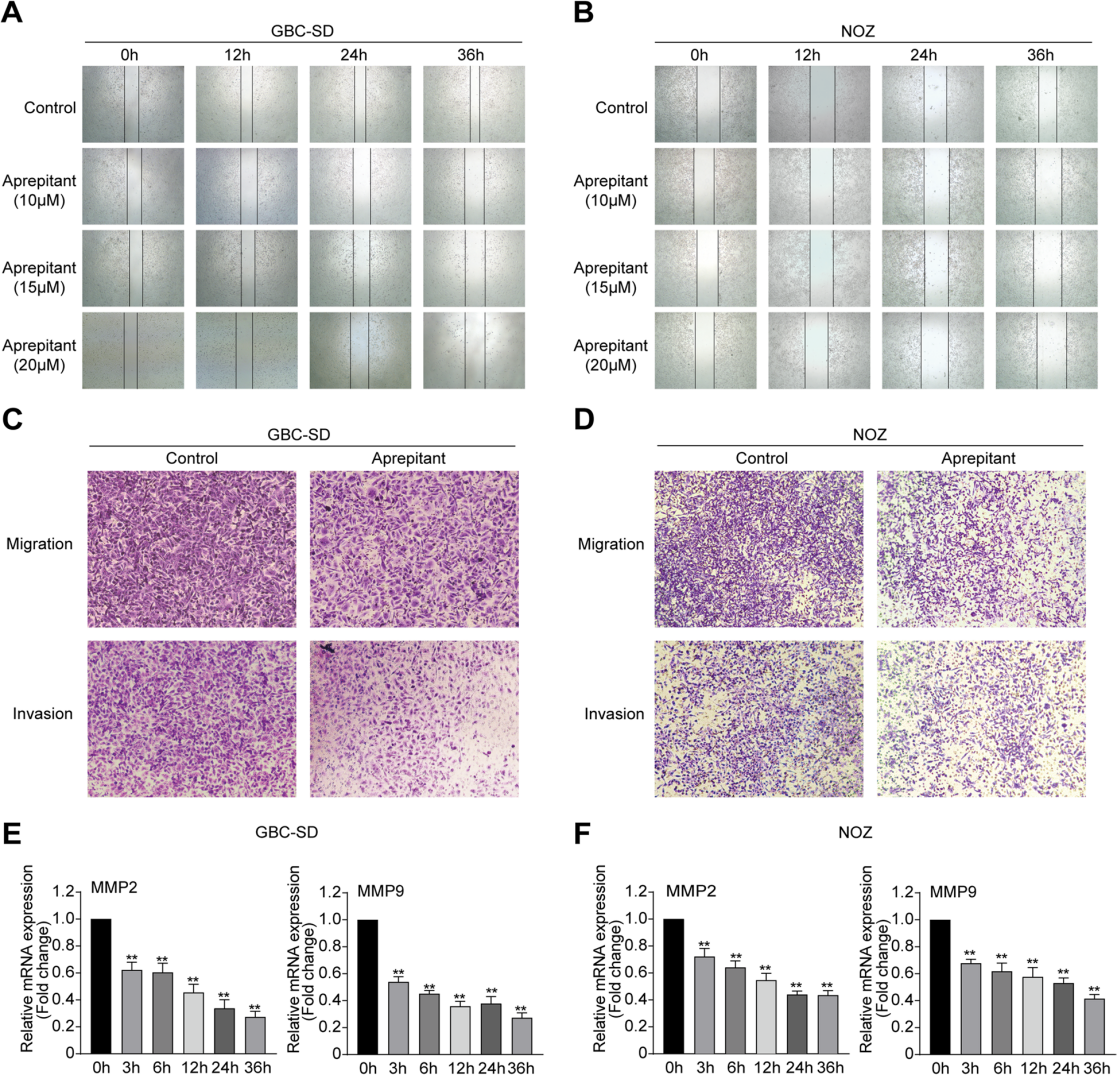
Aprepitant suppressed the migration and invasion in GBC-SD and NOZ cells. GBC‐SD cells (**A**) and NOZ cells (**B**) were scraped with a pipette tip and then cultured with aprepitant at the indicated concentrations for different times. The scratch wound cell migration assays were detected by microscope. The migration and invasion assay were conducted in GBC-SD cells (**C**) and NOZ cells (**D**) treated with or without aprepitant. The cells that invaded across the membrane of the transwell were stained with crystal violet. The relative mRNA levels of MMP2 and MMP9 were measured by real‐time RT‐PCR in GBC-SD cells (**E**) and NOZ cells (**F**), which were treated with aprepitant for the indicated time. Data are presented as means ± SEM (*n* = 7). One-way ANOVA. **p < 0.01, versus 0 h

Cell migration contributes to tumor invasion and metastasis, thereby accelerating tumor progression and reducing patient survival [28]. Thus, we carried out wounding healing assay to investigate the effect of aprepitant on gallbladder cancer cell invasion in vitro. As the images of wound width showed in Fig. 2A,B, treatment with aprepitant for 0, 12, 24, and 36 h gradually reduced the extent of wound closure, compared with the control group, in GBC‐SD and NOZ cells. Meanwhile, transwell migration and matrigel invasion assays were conducted. The results in Fig. 2C,D showed that compared with the control group, fewer gallbladder cancer cells treated with aprepitant could traverse the membrane. The results suggested that aprepitant was able to efficiently inhibit cell migration and invasion.

The matrix metalloproteinases (MMPs), especially MMP-2 and MMP-9, can degrade extracellular matrix components, which are closely related to the tumor migration and metastasis [29]. For further investigation, we detected the mRNA expression levels of MMP-2 and MMP-9 in GBC‐SD cells treated with aprepitant. As shown in Fig. 2E, aprepitant significantly decreased the expression levels of MMP-2 and MMP-9. Consistent with the previous results, a similar effect was observed in NOZ cells (Fig. 2F).

### Aprepitant induces the apoptosis of gallbladder cancerin vitro

**Fig. 3:**
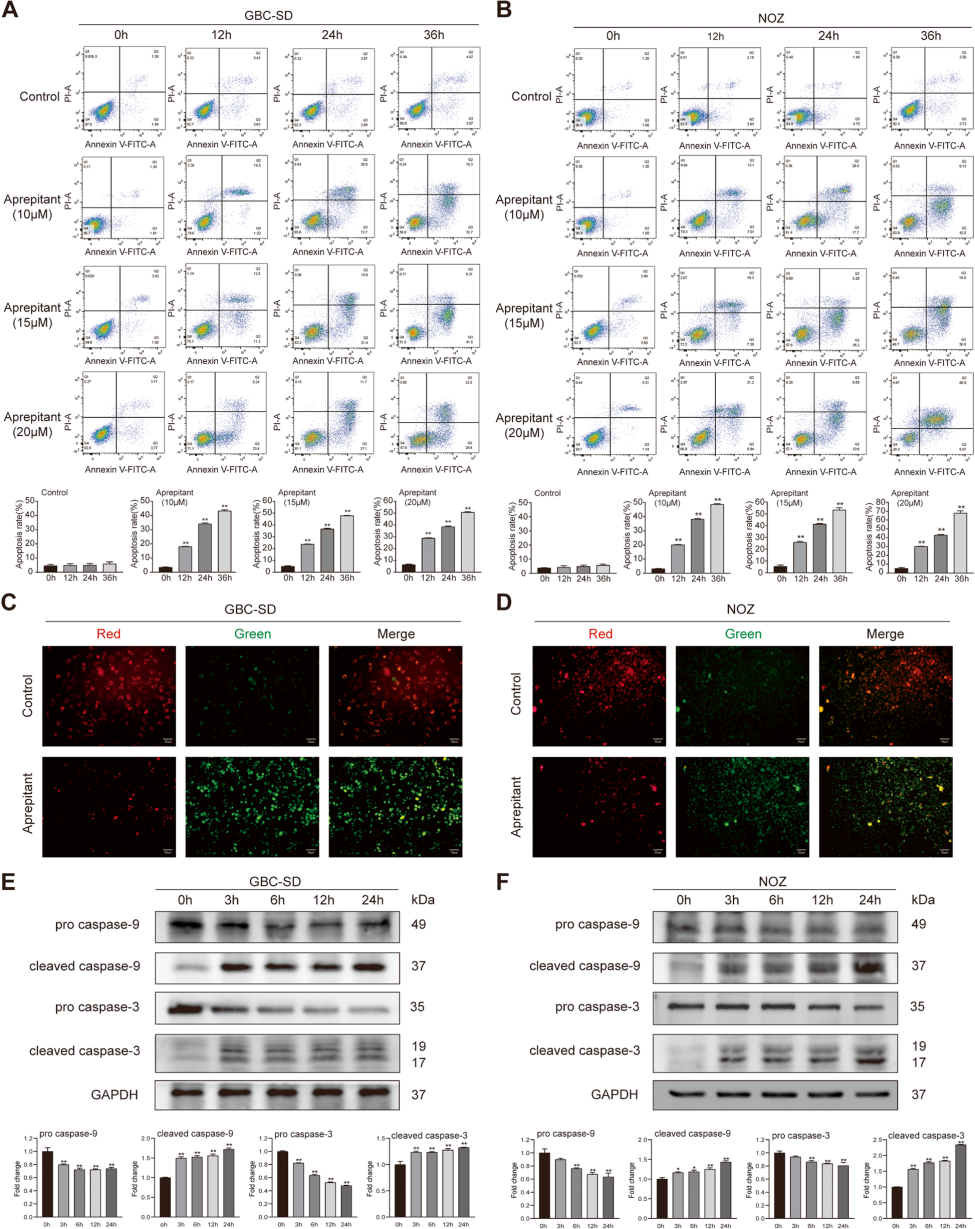
Aprepitant induced the apoptosis in gallbladder cancer cells. After treatment with indicated concentrations of aprepitant for different times, GBC-SD and NOZ cells were stained with Annexin V-FITC/PI. The apoptosis rate of GBC‐SD cells (**A**) and NOZ cells (**B**) was analyzed by flow cytometry. JC-1 staining was performed to detect the changes of mitochondrial membrane potential in GBC-SD cells (**C**) and NOZ cells (**D**). Western blotting detected the expressions of apoptosis-related proteins in GBC‐SD cells (**E**) and NOZ cells (**F**) treated with aprepitant. GAPDH was used as the loading controls. Relative protein expressions were quantified. Data are presented as means ± SEM (*n* = 3). One-way ANOVA. *p < 0.05, **p < 0.01, versus 0 h

We intended to investigate whether apoptosis participates in the cytotoxic effect of aprepitant on gallbladder cancer cells. GBC‐SD and NOZ cells were cultivated with aprepitant in increasing concentrations and stained with Annexin-V/propidium iodide (PI) at 12, 24 and 36 h, so as to evaluate apoptosis by flow cytometry. Consistent with the above antiproliferative effect, we observed that aprepitant significantly augmented the apoptosis rate of GBC cells with the prolongation of drug treatment time and the increase of concentration (Fig. 3A). And the similar effect was observed in NOZ cells (Fig. 3B). To explore whether mitochondrial events participated in the apoptosis mediated by aprepitant, JC-1 staining was used to detect the changes of mitochondrial membrane potential. After administration of aprepitant, the red fluorescence intensity of mitochondria decreased and the green fluorescence intensity increased obviously in GBC‐SD and NOZ cells, which suggested the depolarization of mitochondrial membrane potential (Fig. 3C,D). We further checked if caspase-3 and caspase-9 were involved in aprepitant-induced apoptosis. Western blotting showed that aprepitant promoted the sequential splicing of caspase-3 and caspase-9 both in GBC‐SD and NOZ cells (Fig. 3E,F). Altogether, these data proved that aprepitant significantly induced apoptosis of gallbladder cancer cells.

### Aprepitant promotes the inflammation of gallbladder cancerin vitro

**Fig. 4:**
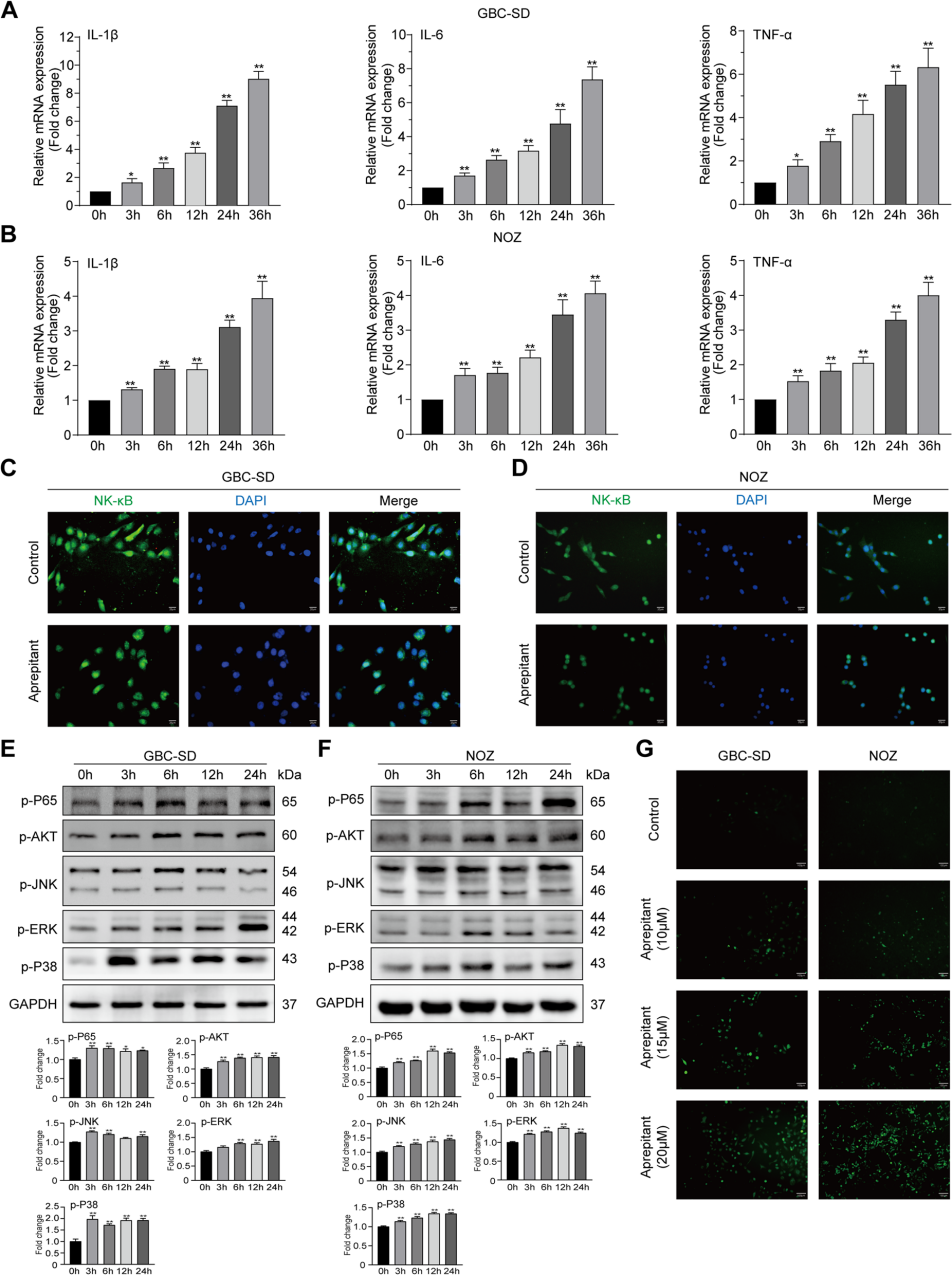
Aprepitant boosted the inflammation of gallbladder cancer in vitro. After administration of aprepitant at 10 μM for 0, 3, 6, 12, and 24 h, the mRNA levels of inflammatory cytokines IL-1β, IL-6 and TNF-α were measured by real‐time RT‐PCR in the GBC‐SD cells (**A**) or NOZ cells (**B**). Immunofluorescence was used to analyze NF‐κB p65 nuclear translocationin in GBC-SD cells (**C**) and NOZ cells (**D**) treated with or without aprepitant (scale bar, 20 μm). The protein expressions of p-P65, p-Akt, p-JNK, p-ERK and p-P38 were detected by western blotting after GBC-SD cells (**E**) and NOZ cells (**F**) were treated with 10 μM aprepitant for different times. GAPDH was used as the loading controls. Relative protein expressions were quantified. After treatment with or without aprepitant, the intracellular ROS production was detected with DCFH-DA in GBC-SD and NOZ cells (**G**). Data are presented as means ± SEM (*n* = 6). One-way ANOVA. *p < 0.05, **p < 0.01, versus 0 h

It is widely known that a large number of inflammatory cytokines might be released along with tumor cell death [30, 31]. Therefore, we investigated whether the cytotoxic effect of aprepitant on gallbladder cancer cells was accompanied by an increase in inflammatory factors. As important components of inflammatory factors, IL‐6, IL‐1β and TNF‐α were measured by qRT-PCR. The results showed that aprepitant significantly increased the mRNA levels of these cytokines in GBC‐SD and NOZ cells (Fig. 4A,B). Notably, NF-κB is the key regulator of inflammatory cytokines [32], so we tested the effect of aprepitant on NF‐κB. The immunofluorescence results indicated that NF‐κB p65 nuclear translocation was markedly induced by aprepitant in gallbladder cancer cells (Fig. 4C,D). Furthermore, the higher expression of phosphorylated NF-κB p65 induced by aprepitant was confirmed at the protein level (Fig. 4E,F).

In tumors, activation of MAPK signaling has positive effect on activity of NF-κB, and its promotion effect always associated with Akt [33, 34]. To clarify the mechanisms underlying aprepitant-induced activation of NF-κB, we assessed the effects of aprepitant on the phosphorylation of MAPK and Akt in GBC-SD and NOZ cell lines. Western blotting revealed that higher levels of p-Akt, p-JNK, p-ERK and p-P38 in GBC‐SD and NOZ cells with aprepitant (Fig. 4E,F). In addition, mounting evidence proved that inflammation is closely linked with ROS production. Accordingly, it was observed that after treatment with aprepitant, intracellular reactive oxygen species (ROS) remarkably accumulated in a dose-dependent manner (Fig. 4G). These findings suggested that aprepitant boosted the inflammation of gallbladder cancer in vitro.

### Aprepitant inhibits GBC xenograft growth

**Fig. 5:**
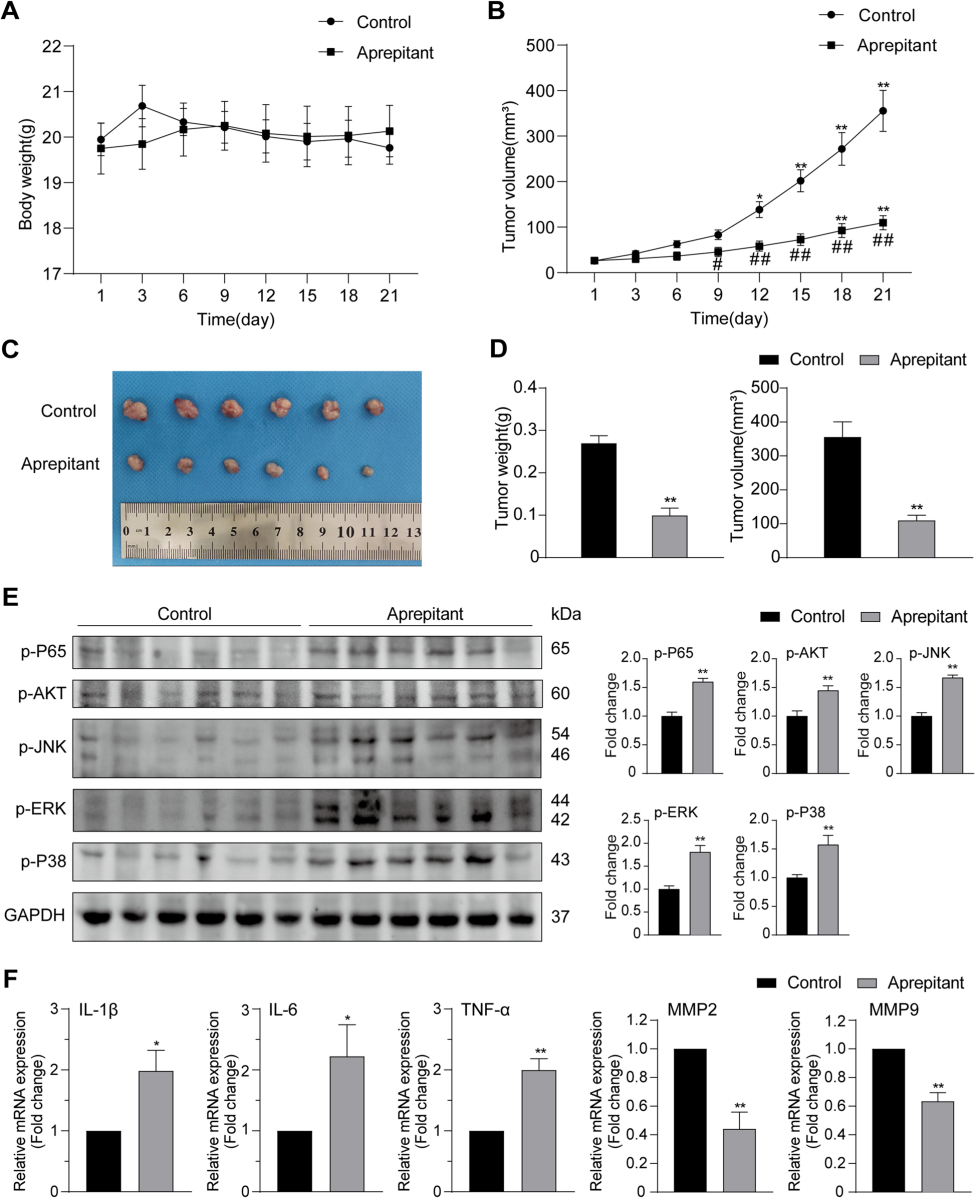
Aprepitant exerted an inhibitory effect on the growth of GBC in vivo. **A** Body weights of nude mice were measured every other day. **B** The tumor volume was measured by vernier caliper for 21 days. **C** Images of tumors obtained from all tumor‐bearing mice were shown. **D** The weight and volume of tumors excised from all mice were recorded. **E** Western blotting was performed to detect the protein levels of p-P65, p-Akt, p-JNK, p-ERK and p-P38 of tumors. **F** The mRNA expressions of IL‐1β, IL‐6, TNF‐α, MMP2 and MMP9 in tumors were determined by real‐time RT‐PCR. Data are shown as means ± SEM (*n* = 6). Unpaired Students t-test and one‐way ANOVA. *p < 0.05, **p < 0.01, versus 0 h. #*p* < 0.05, ##*p* < 0.01 versus Control

Having confirmed a pronounced antitumor effect in vitro, we further verified the effect of aprepitant in vivo by the xenograft nude mice model derived from the GBC‐SD cells. Aprepitant was injected intraperitoneally every other day in the treatment group, while the control group were injected with PBS. No meaningful difference was found in weight change between drug treatment and control groups (Fig. 5A), and none of the treated mice showed serious side effects. Compared to the control group, the tumor of the aprepitant group had obviously slower growth (Fig. 5B) with smaller tumor volume and lighter tumor weight (Fig. 5C,D).

As depicted in Fig. 5E, our western blotting results presented a remarkable increase of p-P65, p-Akt, p-JNK, p-ERK and p-P38 in the aprepitant group. Moreover, we also examined the mRNA expression of IL‐1β, IL‐6, TNF‐α, MMP2 and MMP9. In accordance with the above results, aprepitant augmented the expression of these inflammatory factors and decreased the expression of MMP2 and MMP9 in transcriptional levels (Fig. 5F). In general, aprepitant exhibited a robust inhibitory effect on the tumorigenicity of GBC in vivo.

## Discussion

**Fig. 6:**
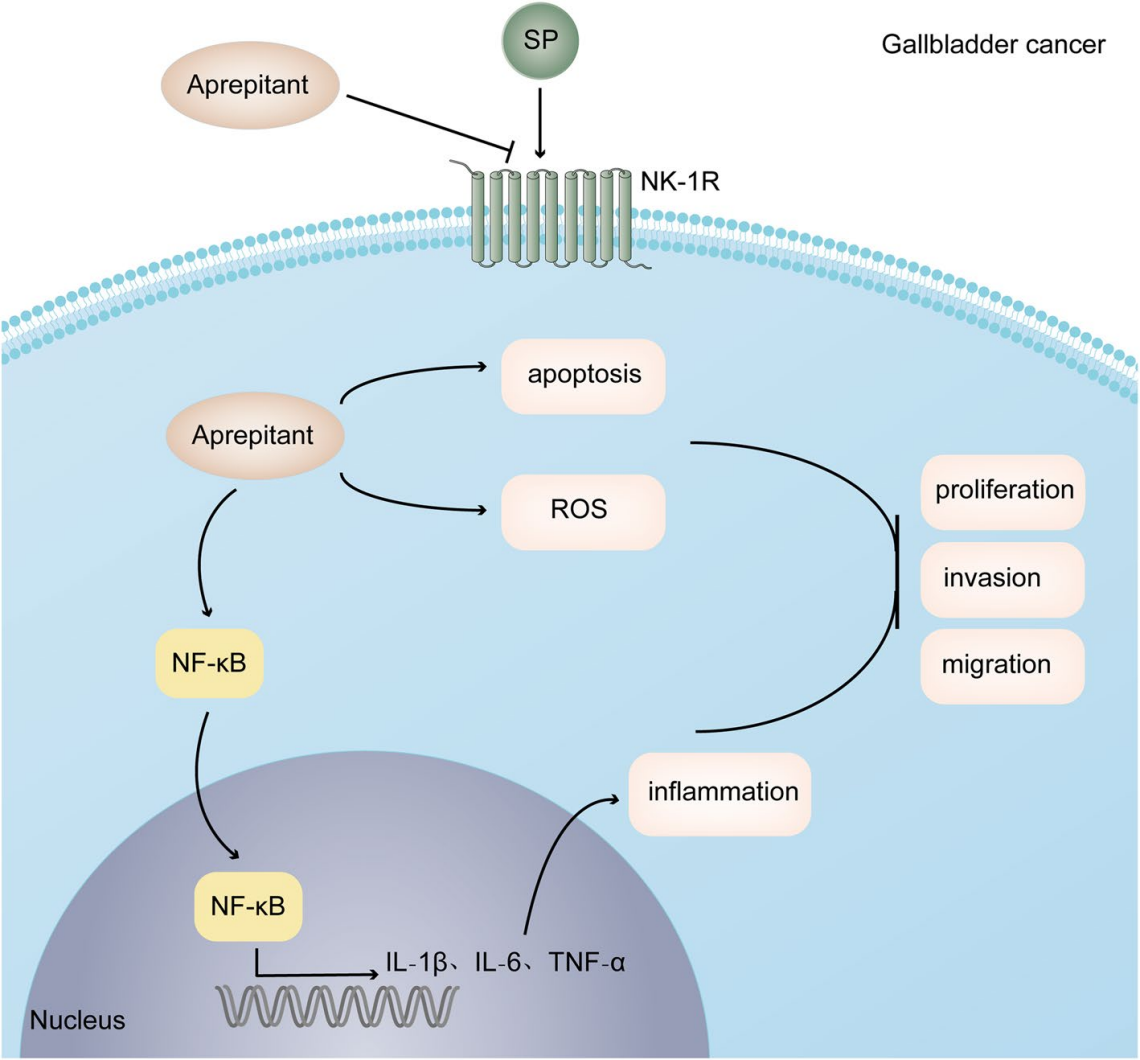
Schematic diagram of the hypothetical mechanism by which aprepitant inhibited the development and metastasis in GBC

In the current study, our results revealed that aprepitant exerts an inhibitory effect on the growth and development of gallbladder cancer in vitro and in vivo for the first time. We showed that NK-1R is markedly expressed in the GBC‐SD and NOZ cells. In line with previous findings about other cancer, aprepitant efficiently inhibited gallbladder cancer cell proliferation in a concentration- and time-dependent manner. Besides, treatment with aprepitant could suppress the migration and invasion of gallbladder cancer cells. The blockage of NK-1R by aprepitant obviously induced the cell apoptosis and inflammation, along with ROS production. Furthermore, we proved that aprepitant inhibits the tumorigenicity of GBC via constructing the xenograft nude mice model. Thus, aprepitant could contribute to the growth inhibition of GBC and may provide an innovative strategy for the gallbladder cancer treatment. Model of the mechanism by which aprepitant inhibited GBC growth is depicted in Fig. 6.

As a highly aggressive malignancy of the biliary tract, GBC is often found at an advanced stage and patients have low survival rate and poor clinical prognosis [4]. Considering that surgical resection is only available for the minority of people, many studies have explored from traditional chemotherapy, radiotherapy, then to immunotherapy for gallbladder cancer. However, the survival benefit of present therapeutic strategy is still very limited, so it is urgent to seek for new treatments [6, 7]. Molecular targeted therapy has the characteristics of strong specificity and little side effects, which helps to change the treatment status of gallbladder cancer [35, 36].

The latest investigations have discovered that the NK-1R is associated with the evolution and progression of tumor. The NK-1R that belongs to the family of G protein-coupled receptors (GPCRs), exerts different biological activities mainly through binding to SP. The activation of NK-1R by SP can cause a series of changes in downstream pathways, which are involved in neurotransmission, inflammation, pain, anxiety and depression, among others [8, 9]. In recent decades, SP/NK-1R system has been discovered to be aberrantly activated in various malignant tumors. For instance, it has been demonstrated that NK-1R expresses highly in melanoma, leukemia, gastric cancer, and breast cancer [13, 17, 19, 37]. In addition, previous studies identified SP as a mitogen to activate NK-1R and thus participate in tumor cell proliferation [38]. For this reason, blocking SP/NK‑1R system with NK-1R antagonists is expected to become a new therapeutic strategy against cancer.

Aprepitant is a selectively high-affinity and non-peptide NK-1R antagonist, which is the first antagonist approved by the FDA to cure chemotherapy-induced nausea and vomiting [21]. Except for the original function, aprepitant have recently been explored for new efficiency that it could inhibit multiple cancers development. M. Munoz et al. showed that aprepitant has broad-spectrum antitumor actions: antiproliferative, antimetastatic and anti-angiogenic effects [22]. To the best of our knowledge, no one has described the effect of aprepitant on gallbladder cancer. Hence, the purpose of our experiments is to investigate whether the clinical drug aprepitant has an inhibitory effect on the gallbladder cancer, which might provide a new strategy for clinical anti-tumor treatment of gallbladder cancer and expand the clinical indication of aprepitant.

Similar to previous findings, our study showed that treatment with aprepitant resulted in the decrease of viability in the gallbladder cancer cells. With the increase of aprepitant treatment time and concentration, the proliferation of GBC‐SD and NOZ cells was obviously inhibited. Based on this, we also calculated that the IC50 values of aprepitant on GBC‐SD and NOZ cells, which were 11.76 and 15.32 µM respectively.

Tumor metastasis involves a series of complicated processes, including cell migration, invasion, adhesion and so on [28]. SP/NK-1R was also suggested to be an important modulator of different cells motility, which takes part in tumor progression and metastasis [39]. It is reported that activating NK-1R by SP could significantly enhance the migration and invasion ability of gastric cancer cell line MKN45 [17]. Considering the high aggressiveness of gallbladder cancer, we tested the influence of aprepitant on gallbladder cancer cell migration and invasion. The results of the wounding-healing assay showed that aprepitant hindered cell migration and the scratch wound healing. Consistently, we proved that cell invasion could be inhibited in vitro by aprepitant via transwell assays with or without matrigel. MMPs, a group of matrix-degrading enzymes, contribute to cancer cells infiltrating into surrounding areas [29]. L. Mou et al. substantiated that the activation of NK-1R distinctly promoted the motility and migration of human glioma cells by up-regulating MMP-2 and MMP14 [40]. Similarly, our qRT-PCR results indicated that the expression levels of MMP-2 and MMP-9 were reduced through blocking NK-1R by aprepitant.

Earlier research described that the anticancer effect of aprepitant depends partly on inducing apoptosis, which seems to be a common mechanism of aprepitant-induced cell death in diverse tumor backgrounds [23, 24, 41]. Apoptosis is a programmed death process initiated by endogenous or exogenous signals, which can effectively inhibit the occurrence and development of tumors. As pointed out by D. Bashash, the cytotoxic effect of aprepitant on acute leukemia cells was mediated through apoptotic [42]. We verified that aprepitant evidently triggered gallbladder cancer cells apoptosis via Annexin-V/PI dual staining method and flow cytometric analysis. With the extending exposure to aprepitant, the cell apoptosis ratio was increased.

When further exploring the underlying impact of aprepitant on gallbladder cancer cells, we discovered that a series of inflammatory reactions occurred after blocking SP/NK-1R system. After cultured with aprepitant for different time, gallbladder cancer cells generated more inflammatory cytokines including IL‐6, IL‐1β and TNF‐α. It is well established that NF-κB functions as is an important regulator of inflammation [32]. Both immunofluorescence and western blotting results suggested that aprepitant promoted the nuclear translocation and phosphorylation activation of NF‐κB in GBC‐SD and NOZ cells. There is mounting evidence that MAPK and Akt signaling pathways are tightly involved in the regulation of NF‐κB transcription activity [31]. Based on the above understanding, our western blotting analysis illustrated that aprepitant increased the protein levels of p-Akt, p-JNK, p-ERK and p-P38 at different time points. Collectively, aprepitant may boost inflammatory responses through activating MAPK and Akt signaling pathways in gallbladder cancer cells. It is common knowledge that ROS elevation plays a prominent part in NF-κB activation and inflammation response [43]. Besides, cell death is partly due to the breakdown of redox state, which is generally balanced by ROS production and scavenging [44]. Given this, our data revealed that compared with the untreated control, aprepitant eminently augmented ROS generation in GBC‐SD and NOZ cells.

Meanwhile, we elucidated the anti-cancer effect of aprepitant on gallbladder cancer in vivo through constructing the xenografted nude mice. The group injected with aprepitant presented distinct reduction in tumor weight and volume. Our findings are consistent with those of others, such as Berger M, who also observed aprepitant had a striking therapeutic effect on HuH6 xenografted nude mice [45]. Significantly, during the experimental period, there was no obvious difference in mice weight change between two groups of mice, which supported the idea that aprepitant had few side effects in vivo. The pharmacologic safety and tolerance of aprepitant has been verified through substantial experiments [15, 46, 47]. The dosage of aprepitant used as antiemetics and antidepressants will not cause serious side effects. Besides, aprepitant is reported to relieve pain [48], which affords greater survival benefitis to GBC patients.

However, the results of our research are relatively limited and further investigations are needed to explore detailed anti-cancer mechanisms of aprepitant. Additional clinical trials are necessary to validate the potential of aprepitant in the treatment of GBC.

In conclusion, we discovered that the clinical drug aprepitant could exert antineoplastic effect on gallbladder cancer by specifically blocking NK1-R both in vitro and in vivo. Our presenting data indicated that aprepitant effectively inhibited the proliferation, migration and invasion of GBC cells, as well as facilitated apoptosis, inflammation and ROS production. These encouraging findings provide evidence that aprepitant might function as a novel and promising anti-cancer agent against GBC.

## Supplementary Information

**Additional file 1: Supplementary figure 1.** Original scanned films. (A) Original scanned films used in mainFigure 3E for caspase-3 and caspase-9, cleaved caspase-3 and cleaved caspase-9 blots. (B) Original scanned films used in mainFigure 3F for caspase-3 and caspase-9, cleaved caspase-3 and cleaved caspase-9 blots. GAPDH was used as the loading controls. **Supplementary figure 2.** Original scanned films. (A) Original scanned films used in mainFigure 4E for p-P65, p-Akt, p-JNK, p-ERK and p-P38 blots. (B) Original scanned films used in mainFigure 4F for p-P65, p-Akt, p-JNK, p-ERK and p-P38 blots. (C) Original scanned films used in mainFigure 5E for p-P65, p-Akt, p-JNK, p-ERK and p-P38 blots. GAPDH was used as the loading controls.

## Data Availability

The original contributions presented in the study are included in the article/supplementary material, further inquiries can be directed to the corresponding author/s.
