# Editorial Expression of Concern: Aprepitant inhibits the development and metastasis of gallbladder cancer via ROS and MAPK activation

**DOI:** 10.1186/s12885-026-16592-0

**Published:** 2026-07-24

**Authors:** Xueyan Cao, Yang Yang, Wei Zhou, Yue Wang, Xue Wang, Xianxiu Ge, Fei Wang, Fangfang Zhou, Xueting Deng, Lin Miao

**Affiliations:** 1 Medical Center for Digestive Diseases, Second Affiliated Hospital, Nanjing Medical University, 121 Jiangjiayuan Road, Gulou District, Nanjing, Jiangsu China; 2 Department of Child Health Care, Children’s Hospital of Nanjing Medical University, Nanjing, China; 3 Burn and Plastic Surgery, Jiangsu University Affiliated Hospital, Zhenjiang, China

**Editorial Expression of Concern: BMC Cancer 23, 471 (2023)**

**https://doi.org/10.1186/s12885-023-10954-8**

The Editors would like to alert readers that concerns were raised regarding similar images in this article. Specifically,

- the Aprepitant 10 µM/12 h and Aprepitant 15 µM/0 h panels in Fig. 2B appear to overlap; and
- the Control/0 h and Aprepitant 10 µM /0 h panels in Fig. 3B appear to overlap

Readers are advised to interpret these results with caution.

Authors Xueyan Cao, Yang Yang, Wei Zhou, Yue Wang, Xue Wang, Xianxiu Ge, Fei Wang, Fangfang Zhou, Xueting Deng and Lin Miao agree with this Editorial Express of Concern.

