# Supplementary material for: Aprepitant inhibits the development and metastasis of gallbladder cancer via ROS and MAPK activation
Source: BMC Cancer. 2023 May 23;23:471. doi: 10.1186/s12885-023-10954-8 (PMC10204192; doi:10.1186/s12885-023-10954-8)
Supplement: Supplementary file 1 — Additional file 1: Supplementary figure 1. Original scanned films. (A) Original scanned films used in mainFigure 3E for caspase-3 and caspase-9, cleaved caspase-3 and cleaved caspase-9 blots. (B) Original scanned films used in mainFigure 3F for caspase-3 and caspase-9, cleaved caspase-3 and cleaved caspase-9 blots. GAPDH was used as the loading controls. Supplementary figure 2. Original scanned films. (A) Original scanned films used in mainFigure 4E for p-P65, p-Akt, p-JNK, p-ERK and p-P38 blots. (B) Original scanned films used in mainFigure 4F for p-P65, p-Akt, p-JNK, p-ERK and p-P38 blots. (C) Original scanned films used in mainFigure 5E for p-P65, p-Akt, p-JNK, p-ERK and p-P38 blots. GAPDH was used as the loading controls. [file 12885_2023_10954_MOESM1_ESM.pdf]

Supplementary figure 1

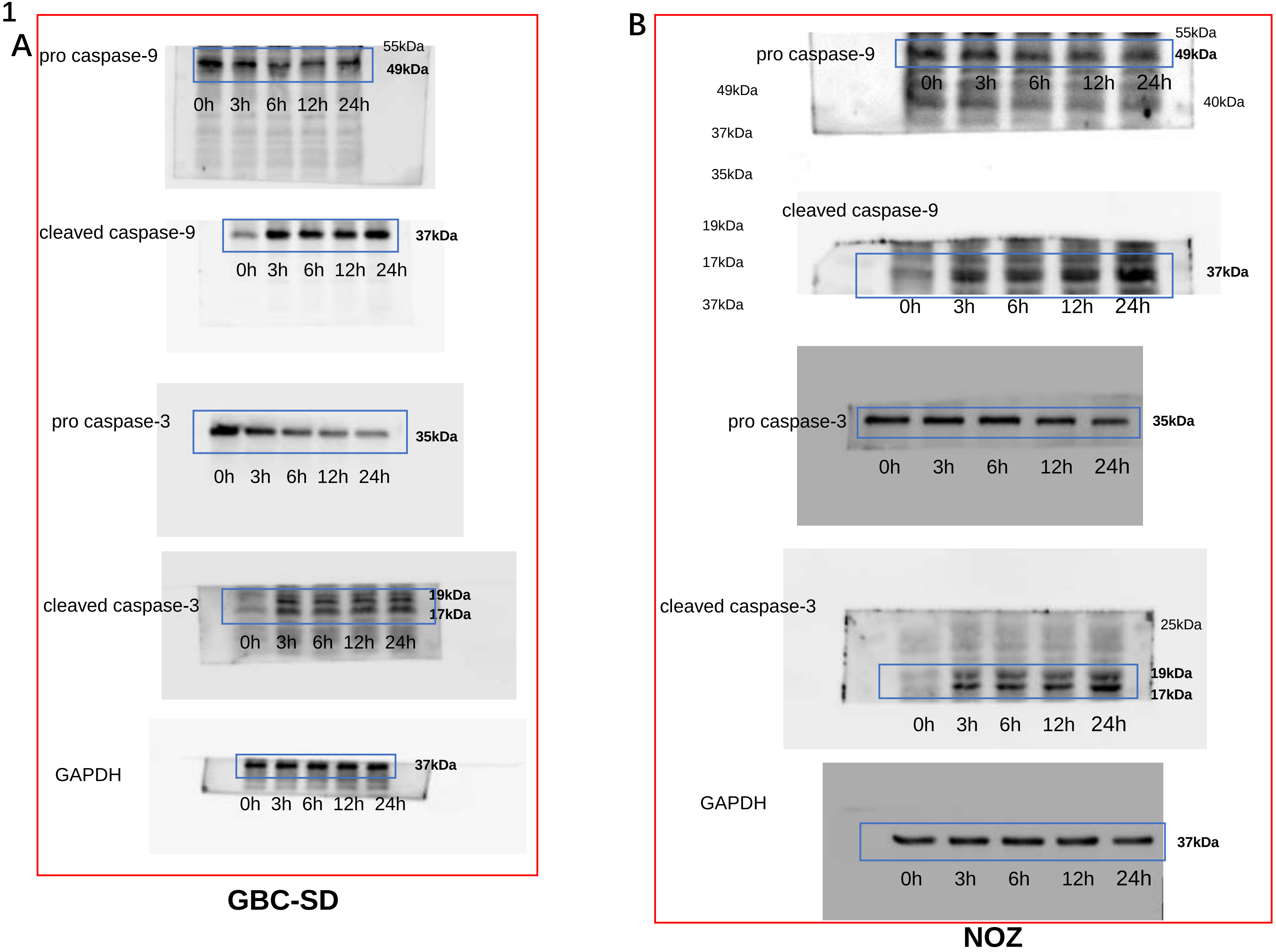

# Supplementary figure 2

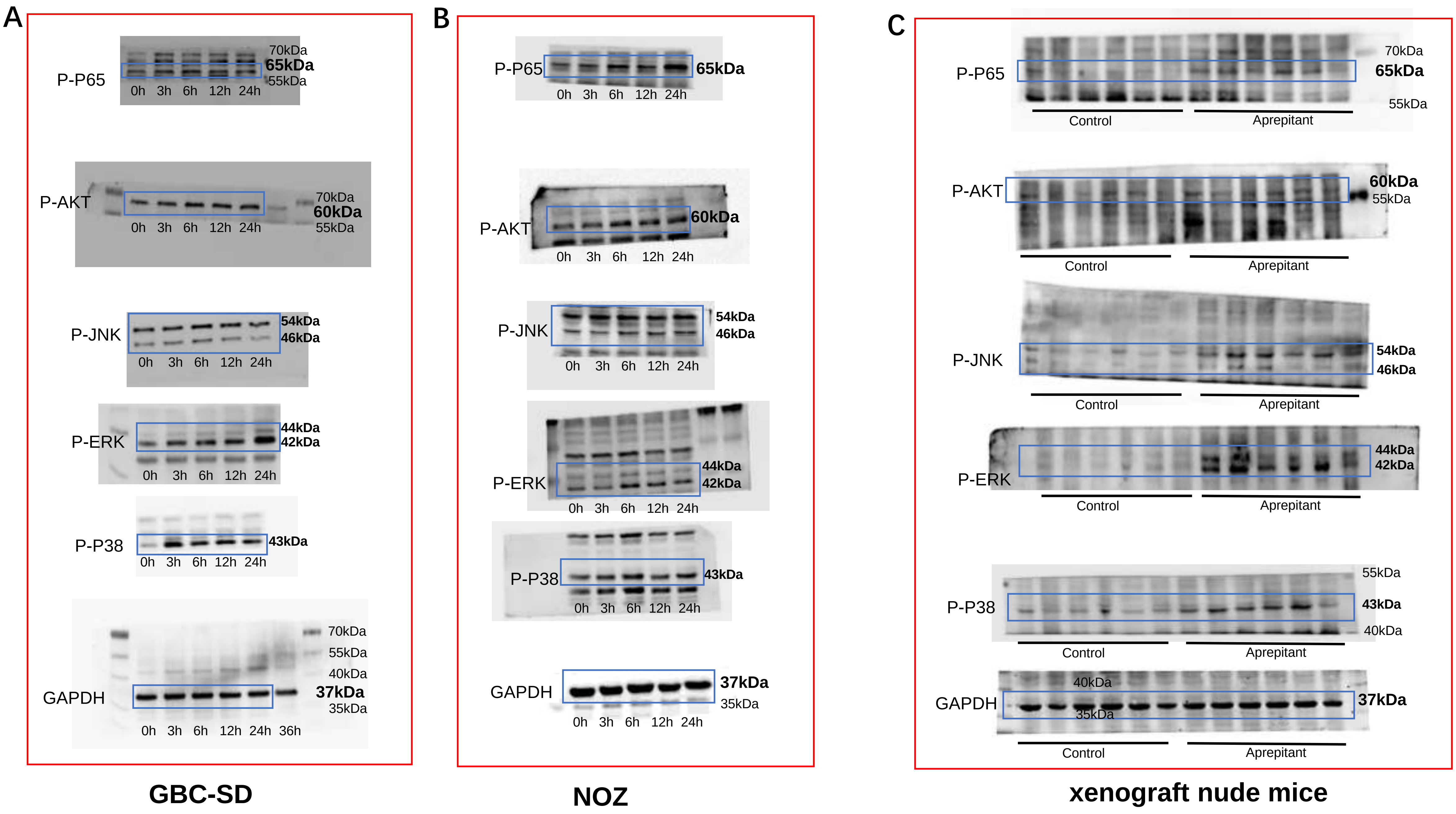

**Supplementary figure 1.** Original scanned films. **(A)** Original scanned films used in mainFigure 3E for caspase-3 and caspase-9, cleaved caspase-3 and cleaved caspase-9 blots. **(B)** Original scanned films used in mainFigure 3F for caspase-3 and caspase-9, cleaved caspase-3 and cleaved caspase-9 blots. GAPDH was used as the loading controls.

**Supplementary figure 2.** Original scanned films. **(A)** Original scanned films used in mainFigure 4E for p-P65, p-Akt, p-JNK, p-ERK and p-P38 blots. **(B)** Original scanned films used in mainFigure 4F for p-P65, p-Akt, p-JNK, p-ERK and p-P38 blots. **(C)** Original scanned films used in mainFigure 5E for p-P65, p-Akt, p-JNK, p-ERK and p-P38 blots. GAPDH was used as the loading controls.
